# Editorial: Text complexity and simplification

**DOI:** 10.3389/frai.2023.1128446

**Published:** 2023-06-13

**Authors:** Liana Ermakova, Valery Solovyev, Grigori Sidorov, Alexander Gelbukh

**Affiliations:** ^1^HCTI, Université de Bretagne Occidentale, Brest, France; ^2^Laboratory ≪Linguistics and Artificial Intelligence≫, Kazan Federal University, Kazan, Russia; ^3^Centro de Investigación en Computación (Center for Computing Research, CIC), National Polytechnic Institute (IPN), Mexico City, Mexico

**Keywords:** text simplification, text complexity, lexical complexity, text clarity, deep learning, simplification benchmarks

## Introduction

Recently, text simplification has raised a lot of interest in the scientific community as numerous texts, including classroom books, scientific articles, legal and financial documents, prove to be too difficult and as such cannot cater to readers' needs. Although the first methods of measuring text complexity were suggested over 70 years ago, the problem is far from being solved. The diversity of languages, text types and genres, as well as their audience, are major challenges for researchers. Despite the significant progress of recent neural models (Sharoff, 2022), many challenges remain unfaced, including the consistency of the long output provided by the models used in a generative context. This Research Topic covers the topic of text complexity and simplification, related notions, resources and methods for English, Portuguese, Spanish, and Russian languages.

## Overview of the Research Topic

Recently, plain and easy language has gained attention as a subject of standardization in many countries. However, even the notions of text clarity, text simplicity, plain language and easy language are problematic. The paper of Vecchiato discusses these four notions and the formal processes of text simplification which should vary accordingly. She highlights that a clear text does not necessarily exclude any ambiguous expression. While much work on automatic text simplification aims to shorten the text, Vecchiato states that a clear text “can be reasonably long if more words are needed to adequately explain a concept.” Vecchiato distinguishes structural, cognitive and development complexity and suggests that a text simplification should integrate the different levels of intelligibility, namely readability, coherence and representability.

The paper (Blinova and Tarasov) develops a model for estimation of the complexity of legal texts in Russian. Several regression and classification methods are compared. Their input are the pre-trained fine-tuning BERT scores and the values of 130 linguistic features. BERT is configured on a tagged textbook corpus of about 10 million words. Such a complex hybrid model is exploited for estimation of the complexity of legal texts in Russian for the first time. It is shown that XGBoost classification model trained on linguistic features and language model predictions achieves the best results. The proposed model shows high results in terms of accuracy of estimation on test data and efficiency in identifying complex legal documents.

The paper (Ivanov) is devoted to the evaluation of the complexity of sentences in Russian. There are 2 datasets in the paper, which are used as the gold standard for the Russian language. One of them contains 75 thousand sentences, the others-−6 million pairs of sentences. The author considers several neural network models, evaluates the complexity of sentences, and shows that fine-tuning pre-trained language models, namely RuBERT, performs slightly better than training a Graph Neural Network. It is fundamentally important that the sentences are aligned in terms of the number of words and the length of words, and it turned out that very high indicators for the accuracy of complexity estimates can be obtained without using these obvious features.

Rosetti and Van Waes discuss stages of text preparation from the perspective of text simplification for second language writers. The authors consider various styles of text rewriting for simplification made by university students. The linear reviser, who prepares the whole text and then starts from the beginning to modify it. The intermittent reviser at each stage comes back to the previous text paragraphs and works with them. The recursive reviser performs revision at smaller portions of texts. The authors analyze dynamics of text production and take into account the expertise of text production in a foreign language. They experimentally study how text simplification training influences text readability in a second language and what is the relationship between the pausing dynamics of writing phases and text readability. Forty-seven Master students participated in their experiment, where they measured text complexity and text cohesion. They also used InputLog of text writing (number of characters types, pauses, cursor position, etc.).

The paper of Dmitrieva et al. presents word alignment from parallel Russian simplification data. This word list is a valuable resource for several practical applications related to text simplification and foreign language teaching. The authors state that not all words are equally easy for learners. The task of monolingual word alignment consists in aligning words or phrases with similar meanings in different sentences in the same language, for example, “to doze off” is more complex than “to fall asleep,” while their meanings are similar. The resulting word list was first scored automatically and then manually evaluated. The size of the list is about 1,400 source lemmas and 800 target lemmas.

Štajner et al. introduced lexical simplification benchmarks for English, Portuguese and Spanish selected and annotated using comparable procedures allowing a fair comparison of lexical simplification systems across three languages. The authors propose simpler synonyms for complex words in 1,153 instances, split across three languages, annotated by 25 crowdsourced workers and validated by a linguist. The dataset is envisioned for evaluation of substitution generation and substitution selection methods. The dataset is limited to one-word terms. Extensive analysis of the dataset is given. The dataset was used for the shared tasks at Text Simplification, Accessibility, and Readability (TSAR).^1^

## Conclusions and perspectives

Despite the recent progress made by large-pretrained models, text simplification remains a challenging problem. Even the terminology related to text complexity and simplification is still not established (Vecchiato). Models for English are dominating in the field, but applications and resources for other languages are emerging. The selection of information and simplification may lead to the alteration of information as well as to potential biases (Ermakova et al., 2023).

Although the goal of text simplification is make it understandable, the question has multiple aspects:

- How much knowledge is required to understand a given text? Is it possible to remove complex terminology? Or should it be explained? (O'Reilly et al., 2019; Ermakova et al., 2023).
- Can we avoid language complexity (too complex words or syntax) with the acceptable level of information distortion? (Ermakova et al., 2023).
- How to reduce second language complexity?

## Author contributions

All authors listed have made a substantial, direct, and intellectual contribution to the work and approved it for publication.

## Footnotes

^1^ https://taln.upf.edu/pages/tsar2022-st/
